# Antegrade Continence Enema Approaches: Outcomes, Lessons Learned and Overall Burden in a Mixed Urban–Rural Population

**DOI:** 10.3390/children13030329

**Published:** 2026-02-26

**Authors:** Brooklyn Ondrea Campbell, Andrew J. Behrmann, Mahmoud Kutmah, Canon Dew, Tara Kempker, Jessica Peuterbaugh, Venkataraman Ramachandran, Yousef El-Gohary, Ahmed I. Marwan

**Affiliations:** 1 University of Missouri School of Medicine, Columbia, MO 65212, USA; bocvgd@umsystem.edu (B.O.C.); ajbgk6@umsystem.edu (A.J.B.); 2 School of Medicine, University of Missouri-Kansas City, Kansas City, MO 64108, USA; mkydx@umsystem.edu; 3 Division of Pediatric Surgery, University of Missouri School of Medicine, Columbia, MO 65212, USA; ccddp9@health.missouri.edu (C.D.); tkempker@health.missouri.edu (T.K.); peuterbaughj@health.missouri.edu (J.P.); ramachandranv@health.missouri.edu (V.R.); yousef.gohary@health.missouri.edu (Y.E.-G.)

**Keywords:** constipation, rural, incontinence, antegrade continence enema, cecostomy, social determinants of health

## Abstract

**Highlights:**

**What are the main findings?**

- MACE and LC were both effective in managing chronic constipation with similar overall complication rates.
- MACE was associated with longer hospital stays, higher 30-day readmission rates, more frequent surgical revisions, and higher healthcare costs compared with LC.

**What is the implication of the main finding?**

- In mixed urban–rural populations, LC may be preferred due to lower healthcare utilization, reduced financial burden, and fewer postoperative complications.

**Abstract:**

**Background**: Antegrade continence enemas (ACEs) are administered by appendicostomy or cecostomy to manage bowel conditions. Cecostomies utilize buttons while appendicostomies utilize the appendix for colonic flushing. This study evaluates the outcomes and overall burden of these procedures in a mixed urban–rural population, highlighting unique social determinants of health (SDoH) and access factors. **Methods**: A retrospective cohort analysis of 31 pediatric patients was conducted at a tertiary academic hospital where 8 underwent a Malone-type ACE (MACE) and 23 underwent a laparoscopic cecostomy (LC) between 2014 and 2024. **Results**: Age at surgery was significantly higher in the MACE group versus the LC group (14.6 vs. 8.1 years). Patients who underwent MACE had longer hospital stays than patients who underwent LC (7.5 vs. 4.5 days, *p* = 0.014) and significantly higher 30-day readmissions (5 vs. 2, *p* = 0.001). Granulation tissue was significant in LC (82.6%) compared to MACE (13.5%, *p* = 0.001). Moreover, need for surgical revision occurred more in the MACE group (25%). Analysis of SDoH revealed that most of the cohort lived in areas with low childhood opportunity and high socioeconomic deprivation, particularly those undergoing MACE. Financially, MACE was associated with substantially higher total, direct, and indirect costs than LC, with the difference in total cost reaching statistical significance. **Conclusions**: In this setting,10-year complication rates were low. This reflects the development of a new dedicated longitudinal bowel management program in mid-Missouri. Functional outcomes at the end of the 10-year period were comparable between both cohorts with the achievement of continence. These findings support tailoring surgical approaches to patient needs.

## 1. Introduction

Constipation and fecal incontinence are common symptoms seen in the pediatric patient population and can have detrimental physical and emotional effects, both on the patients and their families. Most pediatric constipation is idiopathic, but other etiologic causes can include neurogenic bowel, spina bifida, spinal cord injury, pelvic floor dysfunction, chronic fecal retention, anorectal malformation, and Hirschsprung disease, among others [1]. Bowel management programs have become crucial in the management of constipation and fecal incontinence [2]. Bokova et al. demonstrated the successful implementation of bowel management programs, with 70% of patients achieving continence within a two-year period [3].

**Figure 1:**
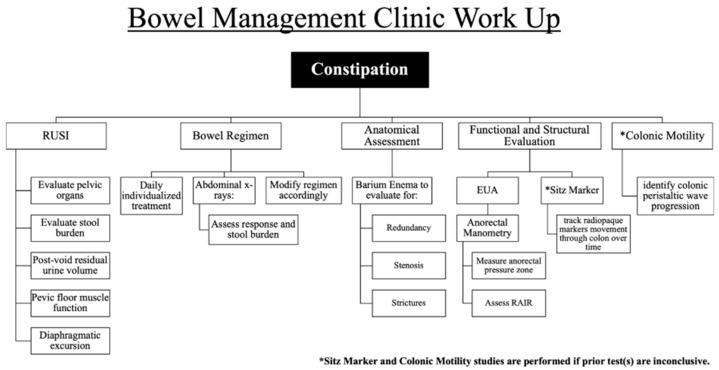
Stepwise diagnostic and management algorithm for pediatric constipation. Initial evaluation includes RUSI to assess pelvic anatomy, stool burden, and pelvic floor function. Daily bowel regimens are adjusted based on clinical response and serial abdominal radiographs. Further assessment with barium enema identifies anatomical abnormalities. If symptoms persist, anorectal manometry and EUA are performed to evaluate anorectal function and reflexes. Colonic motility and Sitz marker studies are reserved for cases with inconclusive findings or suspected colonic dysmotility. Legend: RUSI: rehabilitative ultrasound imaging, EUA: exam under anesthesia, RAIR: rectoanal inhibitory reflex.

Bowel management programs include a structured, multidisciplinary workflow and treatment plans designed to manage all bowel dysfunction by establishing regular, predictable bowel movements [4], with the goal of allowing the patient to participate in normal childhood activities [2]. Typically, treatment plans include dietary modifications, medication regimens, and mechanical interventions. Our institution implemented a structured, multidisciplinary bowel management program in 2022 to evaluate and manage patients with medical and surgical chronic constipation. Our approach is summarized in Figure 1 and includes the innovative use of rehabilitative ultrasound imaging (RUSI) to evaluate the degree of stool burden and rectal morphometry, diaphragmatic excursion, and pelvic floor function to name a few.

At the initial visit to the bowel management clinic, new patients undergo a comprehensive RUSI evaluation [5,6,7]. Standardized assessments are administered, including the pediatric symptom checklist (PSC) [8], the KINDL-R quality of life survey [9], the Krickenbeck classification [10], and an abbreviated version of the Baylor questionnaire [11] to characterize stool consistency and continence. Medication regimens are initiated during this visit. Follow-up patients are evaluated using a standardized intake form, and management is adjusted according to the clinical “zone” assigned based on symptom severity and progress. Our goal is to complete the workup within 4–6 weeks with an individualized patient care plan.

Most cases can be managed non-operatively; however, patients who are refractory to dietary modifications and laxative treatments and who have not achieved a target “green zone” defined by a bowel movement every other day with no soiling/accidents, no pain, and a normal lifestyle within 6–8 weeks are considered for an enema program [12] or offered an antegrade continence approach. However, without proper patient education, enemas in the pediatric population can lead to fear and anxiety [13].

Patients who fall into this category may benefit from surgical intervention for symptom management [14]. Moreover, patients with complex anorectal malformation with urinary fistulae are known to suffer from functional issues due to the absence or malformation of the sphincter complex [15]. Those patients are at a significant risk for fecal incontinence, which is reported to be up to 25% in the literature [16]. The majority of those patients will ultimately need an ACE approach for social cleanliness [17]. The antegrade continence enema was developed to facilitate the delivery of antegrade washouts to induce complete colonic emptying [18]. Enema contents may include normal saline, glycerin, soap, stimulants, and occasionally GoLYTELY [2]. The volume and recipe of the enema is patient-dependent and is adjusted to achieve social cleanliness. Possible approaches for surgical intervention include the Malone antegrade continence enema (MACE) procedure, neo-Malone and laparoscopic cecostomy (LC) [19].

The Malone antegrade continence enema and LC are methods for achieving antegrade access for colonic lavage; however, the difference lies in the access point. The Malone antegrade continence enema creates a catheterizable channel using the appendix as a conduit to the colon [20]. It is classically performed either via an open or a laparoscopic approach with suturing of the appendicular lumen to the skin. A laparoscopic cecostomy directly accesses the cecum through the use of a balloon-tipped button or a Chait cecostomy tube [21] and can be performed either via an open, laparoscopic or a single incision laparoscopic approach [22]. The advantage of MACE is the use of the patient’s own appendix to create a pathway for the enema, whereas cecostomies use an indwelling device to achieve the same goal. However, an LC procedure is easily reversible via removing the balloon device, which in most cases results in the spontaneous closure of the tract and is a simpler procedure than MACE.

Previous studies have documented the effectiveness of both procedures in managing severe constipation and fecal incontinence in the general pediatric population, with specific differences in outcomes between the two. Because there is currently no clear indication to perform one procedure over the other, and due to the potential differences in postoperative outcomes, effects of social determinants of health (SDoH), access, and overall burden associated with either approach and follow-up necessities, it is imperative to evaluate these two approaches, especially in rural patient populations who have less contiguous access to healthcare and a higher burden of SDoH.

This study aims to evaluate outcomes, effects and burden of SDoH on patients who underwent MACE versus LC in a mixed urban–rural patient population following the introduction of our dedicated bowel management program. The University of Missouri Hospital serves a wide rural population as the only children’s hospital in mid-Missouri and is therefore an appropriate center to conduct this research. Understanding these outcomes will not only inform pediatric surgeons’ decisions on which approach to use for the best patient outcome but may also help alleviate the financial burden faced by patients, especially due to limited access and long travel distances to the hospital.

## 2. Materials and Methods

This research was conducted at a single center and is a retrospective cohort analysis of all pediatric patients who underwent Malone antegrade continence enemas (MACEs) or laparoscopic cecostomy (LC) antegrade continence enemas at the University of Missouri between 2014 and 2024. Approval was obtained via the University of Missouri Institutional Review Board (IRB#:2099425). Patients were excluded if they were >18 years at the time of the procedure. Data were collected via electronic medical records (EMRs), manually extracted and stored on a collection sheet. Patients were followed from the time of the procedure until December 2024. The data extracted were medical record numbers, clinic visit dates, birth dates, surgery dates, medication lists, diagnosis codes, diagnostic procedures, operative notes and bowel activity via PSC. Additionally, diagnostic procedures results were reviewed such as anorectal manometry, biopsy, and colonic motility study. Three patients were lost to follow-up due to moving out of state. A total of 31 patients were identified; 8 patients underwent a MACE procedure, and 23 patients underwent LC.

Social determinants of health indices used in this study included the area deprivation index (ADI) [23,24], social vulnerability index (SVI) [25], and childhood opportunity index (COI) [26,27]. ADI was selected to assess socioeconomic status, including factors such as income, education, employment, and public assistance. Scores are reported on a national scale from 1 to 100, with higher scores indicating greater disadvantage, and on a state scale from 1 to 10, with 10 being the most disadvantaged. The SVI was used to evaluate social factors contributing to community vulnerability, including housing, household composition, transportation, and disability. Scores ranged from 0 to 1, with higher values indicating greater vulnerability. The COI was selected to measure opportunity levels related to education, health and environment, and social and economic conditions.

The data were analyzed using various statistical tests depending on the nature of the data. A *t*-test was used for comparing means, while the chi-squared test was used to assess differences in categorical variables. Chi-squared analyses were conducted to assess the relationship between the type of procedure (MACE vs. cecostomy) and several clinical outcomes. The t-test analyses assessed the mean differences between the two groups across various clinical variables. All analyses were performed using SPSS (IBM Statistical Software v.30; Chicago, IL, USA), with a significance level set at *p* < 0.05.

## 3. Results

Demographic and clinical data are summarized in Table 1, surgical outcomes in Table 2, SDoH parameters in Table 3, and a summary of financial data in Table 4.

### 3.1. Demographics

In terms of sex and race, no significant differences were found between the two groups (*p* = 0.11 and 0.16, respectively). Patients who underwent MACE were found to be significantly older than patients who underwent cecostomy (*p* = 0.03). Patients who underwent MACE had a significantly higher BMI at the time of encounter (mean = 26.53) compared with patients who underwent cecostomy (mean = 17.15), (*p* = 0.02) (Table 1).

### 3.2. Surgical Outcomes

However, a significant difference emerged when examining 30-day readmission rates (*p* = 0.001). Patients who underwent MACE had a substantially higher readmission rate (5 out of 8—62.5%) compared with patients who underwent cecostomy (2 out of 23—8.7%). There were no significant differences observed in soiling, surgical site infections (SSI), or ileus (Table 2). Additionally, incisional hernia and parastomal hernia were not observed in either group. Stoma stenosis was only observed in patients who underwent MACE (3 out of 8—37.5%) and was statistically significant compared with LC (0 out of 23—0%) (*p* = 0.01). No significant differences were observed with respect to stomal dehiscence, leaks, perforations, and fecal colonic impaction. Granulation tissue was significantly more common in patients who underwent cecostomy (19 out of 23—82.6%) than in patients who underwent MACE (1 out of 8—12.5%, *p* = 0.001). Revisional surgery was significantly more frequent among patients who underwent MACE (2 out of 8—25%) compared with patients who underwent cecostomy (0 out of 23—0%, *p* = 0.02). Regarding length of stay (LOS), patients who underwent MACE had a significantly longer stay (mean = 7.5 days) compared with patients who underwent cecostomy (mean = 3.68 days) (*p* = 0.01). Blood loss during the procedure was significantly greater in patients who underwent MACE (mean = 55 mL) compared with patients who underwent cecostomy (mean = 6.13 mL), (*p* = 0.03). As expected, and per standard of care and manufacturers’ recommendation, patients who underwent LC (mean = 1.79) had more tube exchanges compared with patients who underwent MACE (*p* < 0.001).

### 3.3. SDoH

Participants were analyzed according to national and state reports on SDoH by address. We found that, overall, 19 out of 31 (61%) of our cohort were categorized as very low to low on the state and national childhood opportunity index (COI). Additionally, nine (29%) had a social vulnerability index (SVI) of medium-high to high. The mean national area of deprivation index was found to be 70 (on scale a of 1–100). Specifically, 22 participants had an ADI score of 60 or greater, and 9 of them had a “high” ADI score of 80 or above, indicating significant levels of socioeconomic deprivation in a portion of the cohort (Table 3). When stratified by procedure, patients undergoing MACE had lower socioeconomic status, with 87.5% having a COI of low or very low, compared with 52% of those undergoing an LC. ADI was different between the two groups, 73.5 and 68 for MACE and LC, respectively. In total, 20 participants (64%) in our cohort were classified as residing in rural areas based on the “Am I Rural?” tool, including 5 participants in the MACE group (63%) and 15 in the LC group (65%).

### 3.4. Finances

Table 4 represents a financial comparison between patients who underwent MACE and those who underwent LC. On average, MACE was associated with substantially higher charges (USD 114,792) compared with LC (USD 47,478), though this difference did not reach statistical significance (*p* = 0.107). The average total cost for MACE was nearly three times higher than for cecostomy, driven by both higher direct costs (USD 23,636 vs. USD 8633, *p* = 0.056) and significantly higher indirect costs (USD 12,361 vs. USD 4315, *p* = 0.022). These differences resulted in a significantly greater total cost for MACE (USD 35,997 vs. USD 12,948, *p* = 0.034). Readmission costs were only reported for the MACE group, adding an average of USD 7067. Although average payments were also higher for MACE (USD 24,725 vs. USD 10,133), this difference was not statistically significant (*p* = 0.196).

## 4. Discussion

This study aims to evaluate long term outcomes, access, SDoH and the overall burden of MACE and LC procedures in a mixed urban–rural patient population following the introduction of our dedicated longitudinal bowel management program. While consistent with the previous literature, our findings uniquely highlight important distinctions in outcomes between MACE and laparoscopic cecostomy in a mixed urban and rural patient population. While both procedures demonstrated low overall complication rates over the 10-year study period, the higher readmission rates, longer hospital stays, and greater likelihood of surgical revisions in patients who underwent MACE suggest a greater burden on healthcare utilization and family resources. These findings imply not only increased direct medical costs but also greater emotional, logistical, and financial strain on families, who may face extended periods away from work or school to provide support during hospitalizations and recovery.

Our findings revealed that those undergoing MACE were approximately 6 years older than those undergoing cecostomy. This is consistent with the previous literature and current clinical practice, as the MACE procedure involves creating a continent stoma using the appendix, requiring a more extensive surgical approach than the percutaneous technique used for cecostomy [28]. As a result, MACE is generally more appropriate for older patients who are better able to tolerate the demands of recovery and ongoing stoma care. It is also frequently performed alongside other complex urological surgeries, such as bladder augmentation or Monti procedures, which are performed in children with severe or refractory neurogenic bowel and bladder dysfunction [29]. This association is supported by findings from Hoy et al., who reported that many patients undergoing MACE also had concurrent urological procedures [29].

MACE has been shown to be associated with a higher rate of complications, such as stomal pain, difficulty with catheterization and stomal stenosis, all of which can be associated with longer hospital stays and readmission that may require trouble shooting or surgical revision [29,30]. Additionally, since MACE utilizes the appendix as a conduit, the postoperative stay could be extended due to education on the use of the MACE stoma, so patients and families learn to manage the system effectively [28,29]. Hoy et al. [29] document that both procedures are comparable in achieving fecal continence (84.6% for MACE and 91.3% with LC. Li et al. [30] reveal that 30% of patients who underwent MACE required additional surgeries due to complications, compared with a 12% revision rate in the LC group.

The significantly higher age at the time of surgery for patients undergoing MACE reflects differences in the underlying indications for the procedure, with neurogenic bowel/bladder dysfunction necessitating more complex management compared with chronic constipation. Our cohort demonstrated similar outcomes, with a 62.5% 30-day readmission rate for mispositioned catheter, deflated Monti when MACE was performed in conjunction, post-op ileus, UTI, post-op nausea and dehydration. The association of creating a catheterizable channel is more complex and may lead to stomal stenosis, leak or prolapse [19,20]. Four patients in our cohort demonstrated difficulty with catheterization of their MACE stoma. Social factors emerged as the main etiology. For example, two patients were away from their home due to factors such as vacations or staying with another parent without catheterization and returned to the office with stoma stenosis. This study’s prevalence of stomal stenosis in patients who underwent MACE may be attributable to the nature of the surgical technique and its long-term effects on stoma function versus complexity and access.

The increased revision rate in patients who underwent MACE, particularly when performed alongside Monti procedures, underscores the need for careful patient selection and long-term follow-up when opting for this approach. In our cohort, three patients underwent a concomitant Monti procedure, with two of them requiring revisions. This did not result in a longer length of stay, which was comparable to other patients who underwent MACE. Indications for revision included urinary leakage and distal detachment of the conduit from the umbilicus. There was a shift in the approach to bowel management at our institution following the development of the bowel management program, with reliance on LC for ACE. This shift was driven by standardized care pathways introduced by the BMP. Complication rates before and after implementation were noted to be similar to those undergoing LC prior to the program, while rates were lower compared with those undergoing MACE. We standardized the workflow, intake process, initial management and subsequent follow-up of all our patients. Moreover, patients were given clear instructions on what is considered a “green zone” and a reasonable time frame to accomplish those goals. Patients who failed to achieve that timeline and functional improvement engaged in a meaningful family-centered discussion towards cecostomy recommendation.

Laparoscopic cecostomy was associated with one incidence of partial dehiscence due to intraoperative findings that were concerning for undiagnosed Crohn’s disease. This patient underwent a diverting ostomy. Granulation tissue was the most common complication observed in the LC group, an expected finding that necessitates additional clinical management with hypertonic saline soaks or chemical cauterization [31]. A single patient underwent an LC with the use of a Chait tube, which was done at the request of the parents. This was prior to the implementation of our program. Another patient’s cecostomy was converted from a laparoscopic to an open approach, as the cecostomy tube could not be passed into the lumen of the cecum.

From a healthcare access perspective, our study further emphasizes the impact of geographic barriers, with an average travel distance of 80 miles for patients undergoing these procedures. This distance presents a significant burden, particularly for patients with a low socioeconomic status, as it may require access to a reliable vehicle, the finances to fuel the vehicle, the ability to take time off work, and/or the availability of someone to provide transportation. This burden, coupled with higher readmission rates in patients undergoing MACE, likely contributes to additional financial and social strain on families, particularly in rural communities with limited healthcare access.

Our analysis of financial strain demonstrates that MACE is associated with significantly higher total, direct, and indirect costs compared with cecostomy, confirming a substantial financial burden on both patients and the healthcare system. The observed discrepancy between costs and payments suggests that our institution may face reimbursement challenges, particularly with high-cost procedures. Although the difference in average charges failed to reach statistical significance (*p* = 0.11) due to a relatively low sample size and variability within the dataset, costs in patients who underwent MACE were roughly three times higher, suggesting clinical and financial significance to children and their families. This is particularly relevant in a mixed urban–rural population, where geographic and logistical factors may amplify the impact of readmissions and hospitalizations. These findings highlight the importance of an individualized approach to procedural selection that considers both clinical outcomes and economic impact. They also suggest possible benefits from proactive strategies to prevent avoidable readmissions, which could improve patient outcomes while reducing unnecessary healthcare spending.

Our analysis was limited to costs associated with postoperative readmissions and did not evaluate preoperative financial burden, baseline healthcare utilization, or neurogenic-specific resource use. Notably, 100% of patients in the MACE cohort had underlying neurogenic bowel or bladder dysfunction. Neurogenic physiology may increase susceptibility to complications such as ileus and UTI, which were documented reasons for readmission in our cohort. However, the admissions analyzed in this study were related to MACE-associated complications and expected postoperative recovery rather than directly attributed to neurogenic status. While baseline complexity may influence overall vulnerability, the financial burden reported here reflects surgery-related readmissions and cannot be independently attributed to patient complexity given the scope of our analysis. Future studies with matched cohorts or risk adjustment may help better distinguish the relative contributions of procedure type and patient physiology.

The socioeconomic challenges faced by the cohort further compound these healthcare access issues. Data on SDoH show that a significant portion of participants come from socioeconomically disadvantaged backgrounds, with limited access to opportunities and moderate levels of social vulnerability. These factors likely contribute to poorer health outcomes and may be particularly relevant when evaluating the long-term effects of the procedures studied. When stratified by procedure, patients who underwent MACE specifically were found to come from areas with a lower COI, with seven out of eight patients who underwent MACE categorized as having low or very low COI. These patients also traveled an average of 95 miles one way to their appointments or surgeries, further underscoring the compounded challenges posed by geographic and socioeconomic barriers. These findings suggest that patients facing both geographic and socioeconomic barriers may experience more difficulty accessing care, which could lead to worse health outcomes and higher healthcare costs, particularly in cases like MACE where readmission rates are higher.

Despite the study’s retrospective, small initial numbers and single-center design, these findings offer valuable insights into optimizing the procedural selection for patients requiring antegrade continence enemas. Future research should focus on strategies to mitigate complications associated with MACE while exploring ways to improve access to care and postoperative management for patients undergoing either procedure. MACE has typically been shown to be the preferred approach in patients with neurogenic bowel and bladder or spina bifida [32]; however, either approach can be performed. Based on the results of this study, in a mixed urban–rural population, cecostomy should be the preferred approach due to lower healthcare utilization and a lower complication profile. Tailoring surgical approaches based on patient characteristics and underlying diagnoses may help reduce the need for revisions and readmissions, ultimately improving long-term patient outcomes.

### Strengths and Limitations

This study possesses several notable strengths and contributes meaningfully to the current literature by integrating clinical outcomes with social and structural factors. The inclusion of SDoH data shows how socioeconomic disparities can affect procedure choice, access to follow-up care, and the burden of complications. Additionally, the financial metrics in conjunction with the SDoH data add a crucial dimension to understanding the resource utilization and economic impact of each procedure. This study took place over a 10-year period, which strengthens the relevance of these findings by capturing durable trends in morbidity, revision rates, and healthcare access over time.

This study has several limitations that should be considered when interpreting the findings. The sample size of 31 patients limits the generalizability of our results and reduces the statistical power to detect more subtle differences between the MACE and LC groups. As a result, this study is at an increased risk of Type II errors, particularly for financial outcomes, and a larger cohort may reveal that these differences reach statistical significance. However, this is the initial experience identified by our group, and, since the implementation of our program, there have been a significant number of additional procedures performed. Additionally, the retrospective design introduces risk of bias, such as selection bias and incomplete data collection, which may affect the accuracy of reported outcomes. These limitations highlight the need for larger, multi-institutional studies in rural populations.

## 5. Conclusions

In this retrospective, single-institution study, we evaluated postoperative outcomes between MACE and laparoscopic cecostomy procedures in a pediatric population at a tertiary academic hospital in a mixed urban-rural setting. While both procedures were effective in managing bowel dysfunction, we found no significant differences in overall complication rates. However, MACE was associated with a longer hospital stay, higher readmission rates, an increased need for surgical revisions, and a higher overall cost, suggesting a greater burden on healthcare utilization and family resources. These findings are particularly relevant in rural communities, where access to specialized care is already limited, and the financial strain of travel and extended hospitalizations can significantly impact families. Moving forward, efforts should focus on optimizing patient selection for each procedure, improving long-term postoperative management, and identifying strategies to minimize healthcare burdens. Future multi-center prospective studies are needed to validate these findings and explore interventions that enhance outcomes while reducing financial and logistical challenges for pediatric patients and their families.

## Figures and Tables

**Table 1:** Cohort demographics.

Demographic Factor	MACE (*n* = 8)	Cecostomy (*n* = 23)	*p* Value (<0.05)
Age in Years (mean, range)	14.6 (7–28)	8.1 (2–16)	0.028
Race			
White (%)	8 (100)	19 (82.6)	0.16
Black (%)	0 (0)	4 (17.4)	
Ethnicity			
Hispanic/Latino (%)	0 (0)	0 (0)	
Non-Hispanic/Latino (%)	8 (100)	23 (100)	
BMI at Encounter (avg)	26.5	17.1	0.02
Type of Cecostomy			
Single Incision Mini/Mickey (%)	-	15 (65.2)	
Mini/Mickey (%)	-	7 (30.4)	
Laparoscopic		6	
Open		1	
Chait (%)	-	1 (4.3)	
Laparoscopic	-	1	
Diagnosis			
Neurogenic Bowel/Bladder (%)	8 (100)	2 (8.7)	
Myelomeningocele/Spina Bifida (%)	5 (62.5)	2 (8.7)	
Spinal Cord Injury (%)	2 (25)	0 (0)	
Anorectal Malformation (%)	0	2 (8.7)	
Colonic Dysmotility (%)	0	1 (4.3)	
Cerebral Palsy (%)	0	3 (13)	
Chronic Constipation (%)	1 (12.5)	16 (69.6)	

**Table 2:** Surgical outcomes by type of procedure.

Outcome	MACE (*n* = 8)	Cecostomy (*n* = 23)	*p* Value (<0.05)
Post-Procedure Outcomes			
Avg LOS, Days (SD)	7.5	4.5	0.01
Readmission (30 days)			
Yes (%)	5 (62.5)	2 (8.7)	0.001
Complications			
Soiling (%)	1 (12.5)	3 (13)	0.83
Male	-	2	
Female	1	1	
Surgical Site Infection			
Superficial (%)	1 (13.5)	1 (4.3)	0.51
Male	-	1	
Female	1	-	
Deep (%)	0	0	
Ileus (%)	1 (13.5)	1 (4.3)	0.51
Male	-	-	-
Female	1	1	-
Incisional Hernia (%)	0	0	-
Parastomal Hernia (%)	0	0	
Stoma Stenosis (%)	3 (37.5)	0	0.01
Male	1	-	
Female	2	-	
Stoma Dehiscence (%)	1 (13.5)	1 (4.3)	0.51
Male	-	-	-
Female	1	1	-
Leak (%)	0	0	-
Perforation (%)	0	0	-
Colonic Fecal Impaction (%)	0	1 (4.3)	0.83
Male	-	1	-
Female	-	-	-
Granulation Tissue (%)	1 (13.5)	19 (82.6)	0.001
Male	1	11	-
Female	-	8	-
Revisions (%)	2 (25)	0	0.02
Male	1	0	-
Female	1	0	-
Blood Loss (ml avg)	55	6.1	
Tube Exchanges (avg)	-	1.1	

**Table 3:** SDOH parameters.

SDOH	Entire Cohort (*n* = 31)	MACE (*n* = 8)	Cecostomy (*n* = 23)
National COI			
Very Low (%)	4 (13)	2 (25)	2 (8.7)
Low (%)	15 (48.4)	5 (62.5)	10 (43.5)
Moderate (%)	8 (25.8)	0	8 (34.5)
High (%)	4 (13)	1 (12.5)	3 (13)
Very High (%)	0	0	0
SVI			
Low (%)	8 (25.8)	0	8 (34.5)
Low-Medium (%)	14 (45.2)	5 (62.5)	9 (39.1)
Medium-High (%)	8 (25.8)	3 (37.5)	5 (21.7)
High (%)	1 (3.2)	0	1 (4.3)
State ADI			
Average	5.5	6.3	5.3
National ADI			
Average	70	73.5	68.2
Distance to Hospital			
Average (miles)	80	95.9	74.5
Rural	20	5	15

COI—childhood opportunity index, SVI—social vulnerability index, ADI—area deprivation index.

**Table 4:** Financial data (USD).

Financial	MACE (*n* = 8)	Cecostomy (*n* = 23)	*p* Value (<0.05)
Charges (avg)	114,792	47,478	0.11
Readmission	25,900	-	
Payments (avg)	24,725	10,133	0.20
Readmission	5284	-	
Cost (avg)			
Direct	23,636	8633	0.06
Indirect	12,361	4315	0.02
Total	35,997	12,948	0.03
Readmission Cost (avg)			
Direct	4453	-	-
Indirect	2614	-	-
Total	7067	-	-

## Data Availability

The data presented in this study are available on request from the corresponding author.

## Abbreviations

The following abbreviations are used in this manuscript:

BMP	Bowel Management Program
RUSI	Rehabilitative Ultrasound Imaging [5,6,7]
EUA	Examination Under Anesthesia
RAIR	Rectoanal Inhibitory Reflex
ACE	Antegrade Continence Enema
MACE	Malone Antegrade Continence Enema
LC	Laparoscopic Cecostomy
PSC	Pediatric Symptom Checklist [8]
LOS	Length of Stay
SSI	Surgical Site Infection
SDoH	Social Determinants of Health [33]
ADI	Area Deprivation Index [23,24]
SVI	Social Vulnerability Index [25]
COI	Childhood Opportunity Index [26,27]
